# Extracorporeal Membrane Oxygenation Education and Training: A Review of Current Practices and Future Directions at a Single Center in Phoenix, Arizona

**DOI:** 10.7759/cureus.118381

**Published:** 2026-10-22

**Authors:** Meriam Avades, Mark Molitor, Melissa Filipiak, Christina Salisbury, Mohammed Elkhwad

**Affiliations:** 1 Neonatology, Phoenix Children's Hospital, Phoenix, USA; 2 Pediatric Surgery, Phoenix Children's Hospital, Phoenix, USA; 3 Extracorporeal Membrane Oxygenation (ECMO), Phoenix Children's Hospital, Phoenix, USA

**Keywords:** ecmo, education, outcomes, simulation, skills and simulation training

## Abstract

Purpose: The purpose of the study is to describe extracorporeal membrane oxygenation (ECMO) training and education practices at our institution and participants’ self-reported comfort with ECMO tasks before and after training sessions.

Background: ECMO is a life-sustaining intervention for critically ill patients with cardiac and/or respiratory failure. ECMO circuit management is complex and requires specialized training. This study describes pediatric and neonatal ECMO education practices at Phoenix Children’s Hospital (PCH).

Methods: We conducted a retrospective analysis of prospectively collected program-evaluation data. The education program ran from 2021 to 2025; anonymous pre- and post-training survey data were available only for 2024-2025. Registered nurses and ECMO primers completed surveys around 2024 water labs, and fellows completed surveys around boot camps in 2024 and 2025.

Results: Survey response rates were 90% (27/30) for spring 2024 water labs and 83% (25/30) for fall 2024 water labs. Among registered nurses and ECMO primers, self-reported comfort was numerically higher after training for managing ECMO emergencies (52% to 74%), ambulating patients (56% to 74%), independently maintaining circuit components (60% to 84%), and escalating pump issues (84% to 96%); all 95% confidence intervals for differences included zero. Fellow item-level response counts varied. Between 2021 and 2025, 273 patients were cannulated onto ECMO at PCH.

Conclusion: In this single-center descriptive evaluation, self-reported comfort was numerically higher after sessions, with imprecise estimates; findings are descriptive and hypothesis-generating. Neither competence nor patient outcomes were measured.

## Introduction

Extracorporeal membrane oxygenation (ECMO) is an advanced intervention used to support patients with life-threatening cardiac and/or respiratory failure, usually when conventional interventions are unsuccessful. A pump and oxygenator provide temporary circulatory and/or respiratory support while the underlying condition is treated [1].

ECMO use in adults expanded during the COVID-19 pandemic [2,3]. With this expansion, the need for standardized training and education programs has rapidly emerged. This paper describes the current practices for ECMO education at Phoenix Children's Hospital (PCH), to our knowledge, the only institution offering programmatic ECMO in pediatric and neonatal patients in the state of Arizona [4].

Importance of ECMO training and education

The use of ECMO is highly specialized and requires proficiency both in the technical operation of the circuit and the management of the patients in which it is used. As with any procedure, ECMO presents certain risks to the patient, including bleeding, equipment failure, and infection. The appropriate use of ECMO within an institution requires highly skilled and well-trained clinicians. Training is therefore relevant to patient safety, clinical decision-making, and interprofessional collaboration.

Optimizing Outcomes

Studies [5,6] have found that patient outcomes are associated with the experience of the healthcare team managing the ECMO circuit.

Interprofessional Collaboration

A multidisciplinary team is typically involved during the management of an ECMO circuit and patient. Incorporating interdisciplinary training, including physicians, nurses, perfusionists, respiratory therapists, and consultants, translates into a collaborative effort during patient management.

To our knowledge, published descriptions of ECMO education come largely from adult programs, and reports of pediatric and neonatal ECMO simulation and education are limited; an eight-year program in a pediatric cardiac intensive care unit (ICU) in Argentina is the closest published comparator [7]. The objective of this study is to describe the ECMO training and education practices at our institution, and to describe participants’ self-reported comfort with ECMO tasks before and after training sessions.

## Materials and methods

Study design and setting

This was a retrospective analysis of prospectively collected program-evaluation data from the ECMO education and training practices at PCH between 2021 and 2025. Survey data were collected only in 2024 and 2025. Survey respondents were registered nurses, ECMO primers (clinicians who prime the circuit and assist with emergency circuit management), and fellows. PCH is the only free-standing children’s hospital in Arizona and, to our knowledge, the only institution offering programmatic ECMO for pediatric and neonatal patients [4]. PCH is an ECMO and extracorporeal life support (ECLS) center within the Extracorporeal Life Support Organization (ELSO). Our institution adopted an ECMO specialist model in which ECMO-trained physicians and bedside registered nurses jointly manage the circuit and patient.

Ethical approval

This project was reviewed by the Phoenix Children’s Institutional Review Board (protocol #22-197), which determined that the project was not human subjects research and waived the requirement for consent. Surveys were anonymous and voluntary.

ECMO education program at PCH

The education and training program at PCH comprises an introductory basic ECMO knowledge course, simulation-based training, and advanced ECMO training for clinical staff.

Basic ECMO knowledge and initial training

The initial training sessions focus on basic ECMO knowledge, including the physiological basis of ECMO, the circuit components, procedures, and indications. The lectures lay a foundation in the basics of ECMO for all clinical staff involved in the care of these patients. A component of this basic training includes a hands-on session about priming a circuit, which allows learners to further understand the circuit components.

Simulation-based training

Simulation-based training is a component of the curriculum. This includes hands-on water labs (sessions using a water-primed circuit and model patient) for various topics, including emergency ECMO initiation and ECLS, troubleshooting, and weaning and decannulation. The emergency ECMO initiation simulation involves a case of sudden cardiac arrest or respiratory failure, requiring immediate action by the learner team.

Simulation sessions use a high-fidelity mannequin as the patient model, connected to a primed ECMO circuit running on a clinical console with live pressure and flow readouts and active alarms. Pressures and alarms displayed during scenarios are generated by the console in response to circuit conditions induced by instructors, such as partially clamping tubing. Scenarios are configured as venoarterial (VA) or venovenous (VV) ECMO. The sessions address circuit priming, cannulation, and team coordination.

Continuous renal replacement therapy (CRRT)-on-ECMO circuit priming was outside the scope of this evaluation, which focused on the ECMO circuit and emergency skills. It is taught separately by the ECMO team and is not currently part of our curriculum.

Advanced ECMO training for clinical staff

To continue providing optimal care to critically ill patients, a focused group of clinicians, including registered nurses, intensivists, and surgeons, participate in advanced ECMO training. The advanced ECMO training often includes periodic refresher courses, peer review sessions, and continuing medical education opportunities. This also includes case-based discussions and mortality and morbidity reviews. At PCH, the ECMO patient care report conference takes place quarterly to discuss relevant cases and provide continuing education.

Additionally, the advanced training is done at the bedside with discussions about the management and monitoring of these patients. All patients cannulated to ECMO will have designated rounds daily with the entire multidisciplinary team. This time is designated to discuss routine management, as well as to troubleshoot and anticipate complications. ECMO rounds are led by the intensive care team, whether neonatal, pediatric, or cardiovascular, with input from primers, surgeons, registered nurses, and consultants.

Finally, competency checklists are used for ECMO specialists, which allow for frequent monitoring and maintenance of competency (Table 1). Checklist scores were not collected or analyzed for this study; the checklist is presented to describe the program and is not an outcome measure in this evaluation.

**Table 1:** Competency checklist for ECMO specialists. Assessment code: 1 = needs improvement: struggles to perform the task consistently; does not show initiative or answer questions. 2 = novice: demonstrates basic understanding; completes tasks with some success; needs guidance for complex tasks; follows along in group scenarios but does not lead or initiate tasks; answers questions with some success. 3 = advanced: solid, reliable understanding of the skill or competency; performs tasks with minimal supervision and meets expectations in most situations; answers questions confidently and asks appropriate questions to advance knowledge; able to lead a group when assigned. 4 = expert: highly skilled and performs tasks with a high level of confidence and competence; handles complex situations and provides guidance to others; in group scenarios, seeks opportunity to lead and acts as coach for others in the group. ECMO: extracorporeal membrane oxygenation

Skills	Date	Evaluated by	Assessment code	Comments
Basic skills: pump assessment, spectrum				
Perform full circuit assessment, verbalize each component of circuit			1 2 3 4	
Identify and define pressure monitors			1 2 3 4	
Verbalize what P2, P3, and Delta P mean in relation to oxygenator health			1 2 3 4	
Discuss the function of “burping”			1 2 3 4	
Demonstrate “burping” the oxygenator by increasing sweep for 30 seconds			1 2 3 4	
Emergency skills: manifold clotted				
Identify signs of manifold clotting			1 2 3 4	
Isolate manifold via stopcocks			1 2 3 4	
Prepare for change out (novice-advanced)			1 2 3 4	
Identify medications likely to precipitate in manifold			1 2 3 4	
Relocate medications in manifold			1 2 3 4	
Replace manifold with primer assistance (novice-advanced)			1 2 3 4	
Replace manifold independently (expert)			1 2 3 4	
Troubleshoot clotted pigtail (advanced-expert)			1 2 3 4	
Identify changes in flow when running without manifold			1 2 3 4	
Emergency skills: pump head exchange				
Identify signs of pump head clotting (pressures, labs, noise)			1 2 3 4	
Verbalize concerns from report: decreased flow, increased clots			1 2 3 4	
Demonstrate “look, listen, feel” to troubleshoot sound: check heater, tanks, pump driver			1 2 3 4	
Communicate to primer			1 2 3 4	
Identify pump driver functioning, pump head needs change			1 2 3 4	
Verbalize emergency vs. urgency: flow? pressures?			1 2 3 4	
Compare risk/benefit of changing pump vs. circuit			1 2 3 4	
Verbalize supplies needed for pump change: pump head, sterile scissors, clamps, ChloraPrep, Plasma-Lyte			1 2 3 4	
Verbalize steps of component change			1 2 3 4	
Change pump head: novice assists primer; advanced leads with primer assistance; expert changes independently and is timed			1 2 3 4	
Clamp off arterial, open bridge, clamp venous			1 2 3 4	
Turn off RPM			1 2 3 4	
Clamp around pump head			1 2 3 4	
Change pump head per level above, airless connections			1 2 3 4	
Unclamp around pump head			1 2 3 4	
Reinitiate RPM, flow through bridge			1 2 3 4	
Perform safety checks while flowing through bridge			1 2 3 4	
Return to bypass (V-B-A), increase RPM to goal flow			1 2 3 4	
Review documentation of procedure			1 2 3 4	
Ambulation on ECMO				
Perform full circuit assessment prior to mobility			1 2 3 4	
Complete mobility checklist			1 2 3 4	
Gather appropriate personnel to assist			1 2 3 4	
Secure cannulas (use securement device and discuss alternatives)			1 2 3 4	
Delegate tasks to each “helper”			1 2 3 4	
Maintain cannula securement throughout ambulation			1 2 3 4	
Ensure safety of patient and circuit during ambulation			1 2 3 4	
Perform circuit assessment after ambulation			1 2 3 4	
Verbal competency				
Identify current quality initiatives			1 2 3 4	
Review documentation of blood products			1 2 3 4	
Review mobility checklist			1 2 3 4	
Discuss anticoagulation monitoring			1 2 3 4	
Verbalize normal lab ranges for bivalirudin anticoagulation			1 2 3 4	

Fellows’ ECMO boot camp

The inaugural boot camp for fellows in cardiology, critical care, neonatal-perinatal medicine, and pediatric surgery was held in the summer of 2024, and a second boot camp for fellows was held in 2025. During the two-day intensive course, fellows received dedicated lectures about the history, physiology, indications, and contraindications to ECMO by the dedicated ECMO training team. They also reviewed patient management and clinical cases with subspecialists in cardiac, neonatal, and pediatric care, including pulmonary hypertension and anticoagulation on ECMO. At the end of day one of boot camp, ECMO primers reviewed circuit components through hands-on priming of a circuit to emphasize the importance of timely activation of the ECMO team in the management of critically ill patients. The boot camp culminated in a hands-on water lab and simulation to review two cases of cardiac and respiratory arrest. Pediatric surgery fellows cannulated model patients while neonatal and critical care fellows managed the circuit with the help of ECMO primers in a true multidisciplinary approach.

Participants and survey administration

Participants and Eligibility

The study population consisted of clinicians participating in ECMO educational activities at PCH during 2024 and 2025. Registered nurses and ECMO primers participating in the spring 2024 and fall 2024 water labs were eligible for survey participation. Fellows in cardiology, critical care, neonatal-perinatal medicine, and pediatric surgery participating in the fellows’ ECMO boot camps were also eligible. Participation was voluntary and anonymous. Completed surveys were included in the analysis; surveys that were not completed were excluded. Because the surveys were anonymous, individual pre-training and post-training responses could not be linked. Registered nurses and ECMO primers were surveyed in 2024 only; fellows were surveyed in 2024 and 2025.

All attendees who chose to complete a survey were included (convenience sample); 30 attendees were eligible for each water lab session, and 10 fellows were eligible for the 2024 boot camp and 15 for the 2025 boot camp. The survey was developed by the ECMO clinical coordinators and was administered electronically immediately before and immediately after each session. For the boot camps, the circuit item was asked before and after day 1 and the emergency item before and after day 2 (Tables 2-5); the wording of some items differed slightly between the pre- and post-surveys. Comfort items addressed managing ECMO emergencies, ambulating patients on ECMO, independently managing circuit components, and escalating pump issues (fellows: ECMO circuits and ECMO emergencies). The spring 2024 and fellows’ items were rated on a 5-point comfort scale (1 = very uncomfortable, 2 = somewhat uncomfortable, 3 = neutral, 4 = somewhat comfortable, and 5 = very comfortable). The fall 2024 items were rated on a 5-point confidence scale with descriptive anchors (Tables 6, 7), on which a rating of 4 indicates confidence with a primer nearby and 5 indicates confidence from extensive experience. “Comfort” is used throughout to refer to both scales. Comfort was reported as the number and percentage (counts only for fellows, because of small and varying denominators) of respondents who rated themselves 4 or 5 (somewhat or very comfortable on the comfort scale; the top two anchors on the fall confidence scale).

**Table 2:** Fellows’ boot camp day one pre-intervention survey design. *5-point comfort scale: 1 = very uncomfortable; 2 = somewhat uncomfortable; 3 = neutral; 4 = somewhat comfortable; 5 = very comfortable. ECMO: extracorporeal membrane oxygenation; VA: venoarterial; VV: venovenous

Domain	Survey item	Response format
Participant information	Date	Date entry (mm/dd/yyyy)
Participant information	Current fellowship specialty	Free-text response
Baseline knowledge	How would you rate your current ECMO knowledge?	3-level scale: novice-limited knowledge and experience; intermediate-basic knowledge and experience; advanced-in-depth knowledge and practical experience
ECMO circuit familiarity	How comfortable are you with Phoenix Children's ECMO circuits?	5-point comfort scale*
VA ECMO	Indications/contraindications for VA ECMO	Understanding scale: limited understanding; basic understanding; advanced understanding
VV ECMO	Indications/contraindications for VV ECMO	Understanding scale: limited understanding; basic understanding; advanced understanding
ECMO management	Daily management of patients on ECMO	Understanding scale: limited understanding; basic understanding; advanced understanding
ECMO procedures	Performing procedures on ECMO	Understanding scale: limited understanding; basic understanding; advanced understanding
Educational needs	What areas would you like more ECMO training in?	Free-text response
Learning objectives	What do you expect to learn about ECMO today?	Free-text response

**Table 3:** Fellows’ boot camp day one post-intervention survey design. ^‡^5-point course-content/hands-on rating: 1 = fair; 5 = excellent. Intermediate response labels were not specified in the source survey. *5-point comfort scale: 1 = very uncomfortable; 2 = somewhat uncomfortable; 3 = neutral; 4 = somewhat comfortable; 5 = very comfortable. ECMO: extracorporeal membrane oxygenation; VA: venoarterial; VV: venovenous; CV: cardiovascular; PPHN: persistent pulmonary hypertension of the newborn; PICU: pediatric intensive care unit

Domain	Survey item	Response format
Participant information	Date	Date entry
Participant information	Specialty	Free-text response
ECMO circuit comfort	PC circuits	5-point comfort scale*
ECMO troubleshooting	Flow issues	5-point comfort scale*
VA ECMO	VA	Understanding scale: basic understanding; advanced understanding; expert understanding
VV ECMO	VV	Understanding scale: basic understanding; advanced understanding; expert understanding
ECMO management	Daily management	Understanding scale: limited understanding; basic understanding; advanced understanding
ECMO procedures	Procedures	Understanding scale: limited understanding; basic understanding; advanced understanding
Course feedback	What did you find most helpful from today's class?	Free-text response
Course feedback	What aspects of this class could be improved?	Free-text response
Course content evaluation	History ECMO	5-point course-content rating^‡^
Course content evaluation	Phys components ECMO	5-point course-content rating^‡^
Course content evaluation	VA/VV ECMO	5-point course-content rating^‡^
Course content evaluation	CV ECMO Mgmt	5-point course-content rating^‡^
Course content evaluation	PPHN ECMO	5-point course-content rating^‡^
Course content evaluation	PICU Mgmt	5-point course-content rating^‡^
Hands-on education	Hands-on water lab w/ ECMO primers	5-point course-content rating^‡^

**Table 4:** Fellows’ boot camp day two pre-intervention survey design. *5-point comfort scale: 1 = very uncomfortable; 2 = somewhat uncomfortable; 3 = neutral; 4 = somewhat comfortable; 5 = very comfortable. ECPR: extracorporeal cardiopulmonary resuscitation; ROTEM: rotational thromboelastometry; ECMO: extracorporeal membrane oxygenation

Domain	Survey item	Response format
Participant information	Date	Date entry (mm/dd/yyyy)
Participant information	What is your current fellowship specialty?	Free-text response
ECMO knowledge	How would you rate your current ECMO knowledge?	3-level knowledge scale: novice-limited knowledge and experience; intermediate-basic knowledge and experience; advanced-in-depth knowledge and practical experience
ECMO emergency experience	Have you ever assisted in an ECMO emergency?	Free-text response
ECMO emergency comfort	How comfortable are you with responding to ECMO emergencies?	5-point comfort scale*
Anticoagulation	Anticoagulation on ECMO	4-level understanding scale: limited understanding; basic understanding; advanced understanding; expert understanding
ROTEM	ROTEM interpretation on ECMO	4-level understanding scale: limited understanding; basic understanding; advanced understanding; expert understanding
Weaning/decannulation	Weaning and decannulation from ECMO	4-level understanding scale: limited understanding; basic understanding; advanced understanding; expert understanding
ECPR/emergencies	ECPR and other ECMO emergencies	4-level understanding scale: limited understanding; basic understanding; advanced understanding; expert understanding
Learning objectives	What do you expect to learn about ECMO today?	Free-text response

**Table 5:** Fellows’ boot camp day two post-intervention survey design. *5-point comfort scale: 1 = very uncomfortable; 2 = somewhat uncomfortable; 3 = neutral; 4 = somewhat comfortable; 5 = very comfortable. ECMO: extracorporeal membrane oxygenation; ECPR: extracorporeal cardiopulmonary resuscitation

Domain	Survey item	Response format
Participant information	Date	Date entry (mm/dd/yyyy)
Participant information	What is your current fellowship specialty?	Free-text response
ECMO emergency management	How comfortable are you with patient management and response to ECMO emergencies?	5-point comfort scale*
ECPR activation	How comfortable are you with activating ECPR?	5-point comfort scale*

**Table 6:** Fall 2024 water lab pre-intervention survey design. ^†^5-point confidence scale: 1 = “I’d prefer not to, can I go on break now?”; 2 = “I need a lot of help because I’m so new at this.”; 3 = “I can do it, but someone else needs to call the shots.”; 4 = “I have no fear when my primer is near!”; 5 = “I’m a pro! I’ve done it a million times!”

Domain	Survey item	Response format
Confidence assessment	Escalating pump issues to primer	5-point confidence scale^†^
Confidence assessment	Independence during component maintenance	5-point confidence scale^†^
Confidence assessment	Assisting with a component change?	5-point confidence scale^†^
Confidence assessment	Recognizing component failure?	5-point confidence scale^†^

**Table 7:** Fall 2024 water lab post-intervention survey design. ^†^5-point confidence scale: 1 = “I’d prefer not to, can I go on break now?”; 2 = “I need a lot of help because I’m so new at this.”; 3 = “I can do it, but someone else needs to call the shots.”; 4 = “I have no fear when my primer is near!”; 5 = “I’m a pro! I’ve done it a million times!” VAD: ventricular assist device

Domain	Survey item	Response format
Confidence assessment	Escalating pump issues to primer	5-point confidence scale^†^
Confidence assessment	Independence during component maintenance	5-point confidence scale^†^
Confidence assessment	Assisting with a component change?	5-point confidence scale^†^
Confidence assessment	Recognizing component failure?	5-point confidence scale^†^
Confidence assessment	VAD maintenance?	5-point confidence scale^†^

Survey design: The instruments were developed for program evaluation and were not validated; they are provided in Tables 2-9.

**Table 8:** Spring 2024 water lab pre-intervention survey design. *5-point comfort scale: 1 = very uncomfortable; 2 = somewhat uncomfortable; 3 = neutral; 4 = somewhat comfortable; 5 = very comfortable. ECMO: extracorporeal membrane oxygenation

Domain	Survey item	Response format
Participant information	Form date	Date entry (mm/dd/yyyy)
ECMO experience	How long have you been doing ECMO?	Free-text response
Ambulation	How comfortable do you feel ambulating ECMO?	5-point comfort scale*
ECMO emergencies	How comfortable are you handling ECMO emergencies?	5-point comfort scale*
ECMO emergencies	What ECMO emergency scares you the most? Why?	Free-text response
ECMO maintenance	What ECMO maintenance scares you the most? Why?	Free-text response

**Table 9:** Spring 2024 water lab post-intervention survey design. *5-point comfort scale: 1 = very uncomfortable; 2 = somewhat uncomfortable; 3 = neutral; 4 = somewhat comfortable; 5 = very comfortable. ECMO: extracorporeal membrane oxygenation

Domain	Survey item	Response format
Participant information	Form date	Date entry (mm/dd/yyyy)
Ambulation	How comfortable do you feel ambulating ECMO?	5-point comfort scale*
Ambulation learning	What did you learn ambulating ECMO today?	Free-text response
ECMO emergencies	How comfortable are you handling ECMO emergencies?	5-point comfort scale*
ECMO emergency learning	What did you learn from today’s ECMO emergency?	Free-text response
Family education	How was your ECMO knowledge (family feud)?	Free-text response
Water lab feedback	Any other water lab feedback?	Free-text response

Statistical analysis

Survey responses were summarized descriptively as frequencies and percentages. Because pre- and post-training surveys were anonymous and could not be linked, responses were treated as independent samples rather than paired observations. No hypothesis tests or p-values were calculated. We report 95% Wilson confidence intervals (CIs) for single proportions and Newcombe hybrid score CIs for differences in proportions (post minus pre), treating pre- and post-surveys as independent samples. These exploratory intervals are not adjusted for multiple comparisons or possible non-independence of respondents. No a priori sample-size calculation was performed. The analysis included available completed surveys.

Potential confounders

Because this was a descriptive evaluation without linked individual-level pre/post data, potential factors influencing self-reported comfort-including prior ECMO experience, professional role, home ICU, years in practice, previous simulation exposure, intervening ECMO case exposure, staff turnover, and training-session composition-were not measured or adjusted for. Role and home ICU were not collected, so respondents could not be stratified and non-response bias could not be assessed.

## Results

Survey participation

Formalized survey data were available only for 2024 and 2025; no survey data were available from 2021 to 2023. Results are reported for registered nurses and ECMO primers in 2024 and fellows in 2024 and 2025; nurses and primers were not surveyed in 2025. Response rates were 90% (27/30 eligible) for spring water labs and 83% (25/30 eligible) for fall water labs. For fellows, item-level response counts in 2024 ranged from 4 to 10 of 10 eligible; all 15 eligible fellows completed each reported item at both time points in 2025. Because role and home ICU were not collected, non-response bias could not be assessed.

Comfort levels after training

During water labs held in the spring of 2024, 27 registered nurses and ECMO primers completed pre-training and post-training surveys (Figure 1). Comfort in handling ECMO emergencies increased from 52% of respondents (14 of 27; 95% CI 34%-69%) reporting comfort before training to 74% (20 of 27; 95% CI 55%-87%) after training. Comfort in ambulating patients on ECMO increased from 56% (15 of 27; 95% CI 37%-72%) before training to 74% (20 of 27; 95% CI 55%-87%) after training. The differences (post minus pre) were +22 percentage points (95% CI −3 to +44) for emergencies and +19 (95% CI −7 to +41) for ambulation.

**Figure 1:**
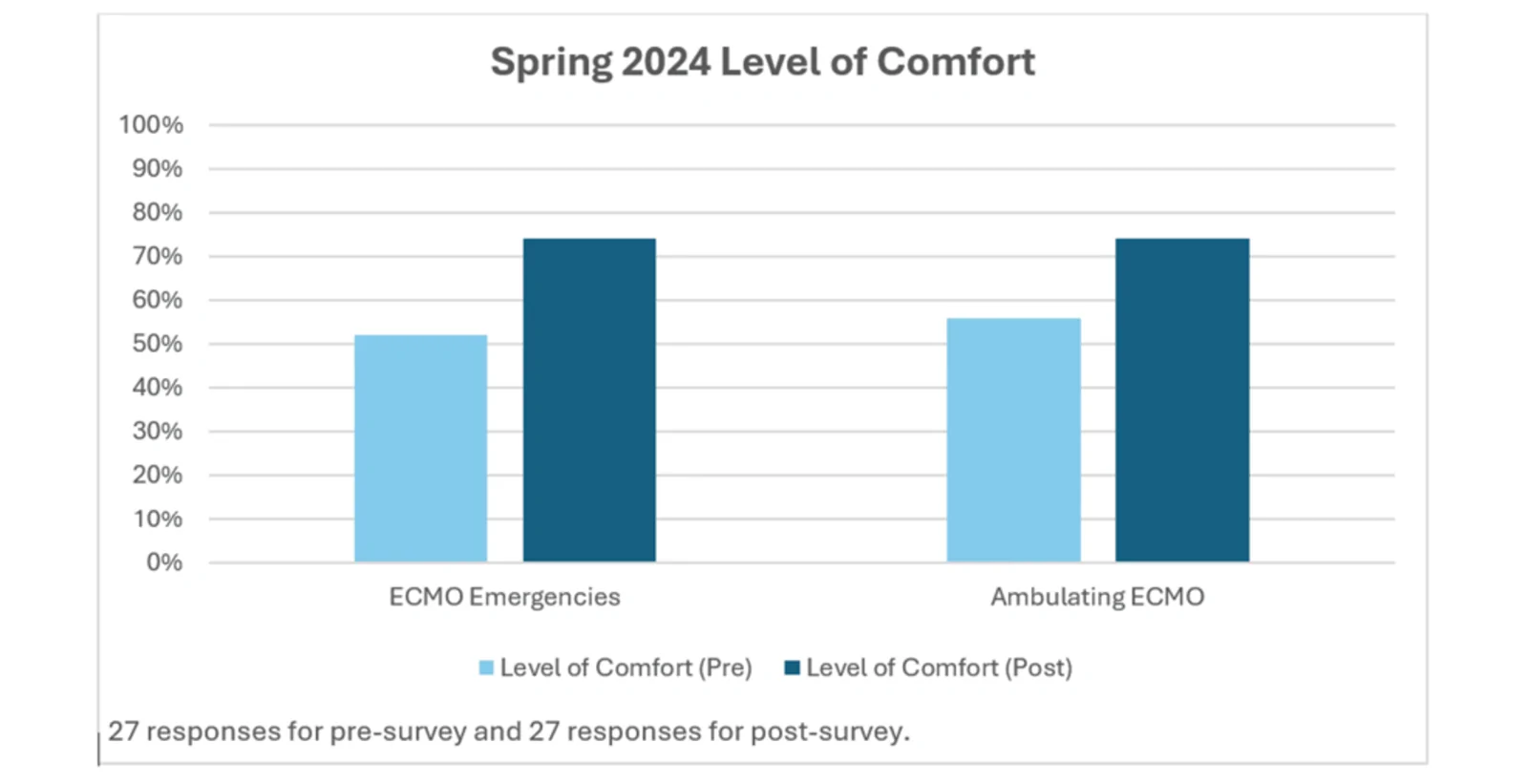
Level of comfort in handling ECMO emergencies and ambulating patients on ECMO among registered nurses and ECMO primers, spring 2024 (n = 27 pre-survey, n = 27 post-survey). ECMO: extracorporeal membrane oxygenation

Similarly, during water labs held in the fall of 2024, 25 registered nurses and ECMO primers completed pre-training and post-training surveys (Figure 2). Comfort with independently managing circuit components increased from 60% of respondents (15 of 25; 95% CI 41%-77%) before training to 84% (21 of 25; 95% CI 65%-94%) after training. Comfort with escalating pump issues increased from 84% (21 of 25; 95% CI 65%-94%) before training to 96% (24 of 25; 95% CI 80%-99%) after training. The differences were +24 percentage points (95% CI −1 to +46) for circuit components and +12 (95% CI −6 to +31) for escalation.

**Figure 2:**
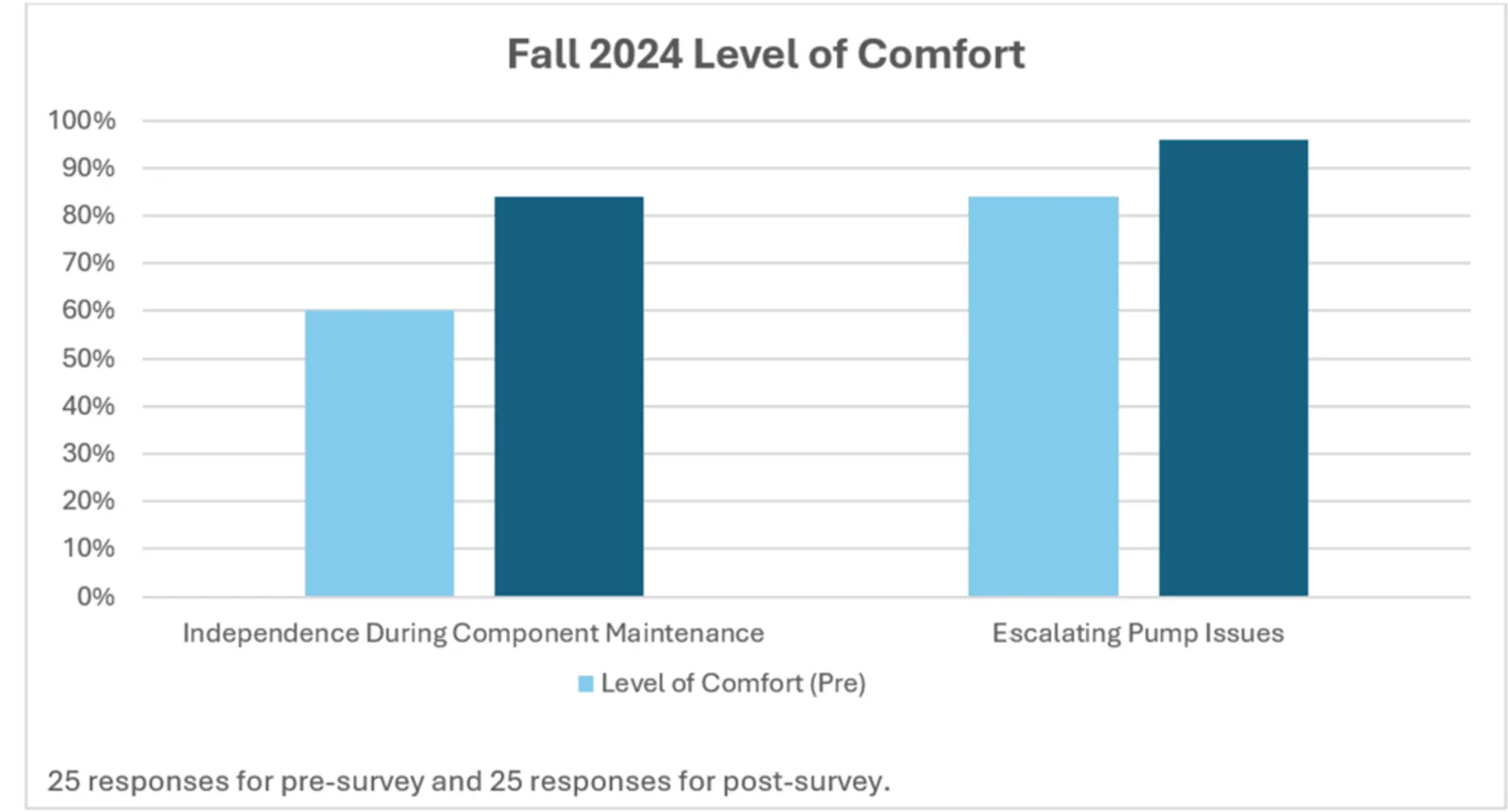
Level of comfort in independently maintaining ECMO components and escalating pump issues among registered nurses and ECMO primers, fall 2024 (n = 25 pre-survey, n = 25 post-survey). ECMO: extracorporeal membrane oxygenation

Fellows’ boot camp

In 2024, 10 fellows were eligible for boot camp. Respondent counts varied by item and time point (circuit item: 10 before and 6 after; emergency item: 6 before and 4 after). Because denominators were small and differed across time points, results are presented as separate descriptive distributions rather than paired responses. On the comfort scale, comfort with ECMO circuits was reported by 4/10 before and 4/6 after the boot camp, and comfort with ECMO emergencies by 2/6 before and 3/4 after. In 2025, comfort with ECMO circuits was reported by 3/15 before and 7/15 after, and comfort with ECMO emergencies by 3/15 before and 8/15 after. These estimates are imprecise. In 2024, free-text responses from a small number of fellows identified the hands-on water lab as a valued component of training; this qualitative observation should not be interpreted as unanimity among all eligible fellows.

Program activity

Between 2021 and 2025, 273 patients were cannulated onto ECMO at PCH, an average of approximately 55 circuits per year across the three ICUs. In 2025 alone, 71 patients were cannulated.

## Discussion

Principal findings

ECMO is a technically challenging intervention that requires a trained multidisciplinary team that can manage the daily complexity of the circuit, in addition to recognizing complications and responding with efficiency. With the expansion of the use of ECMO after the COVID-19 pandemic, the need for a standardized training and education program arose. Our institution developed a curriculum to address the need for experienced and competent specialists.

Based on anonymous surveys completed before and after hands-on ECMO training sessions in 2024, self-reported comfort in managing ECMO emergencies, ambulating patients on ECMO, independently maintaining components, and escalating issues was numerically higher after training among registered nurses and ECMO primers, although all 95% CIs for the differences included zero. In 2024, fellows unanimously identified the hands-on water lab as the most valued component of training.

Comparison with existing literature

Our findings are consistent with other single-center experiences describing structured ECMO education programs. In an eight-year single-center experience in Argentina, a formal theoretical-practical curriculum with simulation and annual maintenance training similarly improved long-term staff comfort and confidence in managing ECMO patients [7]. Adult programs that have adopted nurse-run or multidisciplinary ECMO specialist staffing models have reported survival and complication rates comparable to those of other staffing models [8,9]. We collected no cost or outcome data, so these reports provide context but do not support the specialist model used at our institution [10].

In our surveys, the proportion of registered nurses and ECMO primers reporting comfort (a rating of 4 or 5) was numerically higher after training on all four items measured, with absolute differences ranging from 12 to 24 percentage points, but all four 95% CIs included zero. The largest gains were in handling ECMO emergencies (52% to 74%) and independently managing circuit components (60% to 84%). The smaller gain in escalating pump issues (84% to 96%) began from a high baseline, which may reflect a ceiling effect. Fellows in the boot camp showed the same direction of change for comfort with ECMO circuits (4 of 10 to 4 of 6 in 2024; 3 of 15 to 7 of 15 in 2025) and ECMO emergencies (2 of 6 to 3 of 4 in 2024; 3 of 15 to 8 of 15 in 2025), but these estimates rest on 4 to 15 respondents per item and should be interpreted as preliminary; none of the fellow items had a 95% CI for the difference that excluded zero.

These findings should be read as differences in self-reported comfort between pre- and post-session respondents, not as measured competence. Comfort is an early and modest educational outcome, and it may not reflect performance during an actual ECMO emergency. Because the surveys were anonymous, could not be linked between individuals, and were analyzed only descriptively, we cannot attribute the observed differences to training alone. The design is similar to that of the Argentinian program, which also used an anonymous, voluntary, self-administered survey to assess perceived confidence in ECMO skills; however, that survey assessed long-term perception, whereas ours captured comfort only immediately before and after each session and therefore cannot speak to retention [7].

Fellows in the 2024 boot camp most valued the hands-on water lab. This is consistent with a randomized trial of simulation-based training for critical care fellows managing ECMO emergencies [10] and with our group’s experience in which case-based training and workshops were the most useful components of ECMO education [11]. Because individual specialists may encounter ECMO emergencies only rarely, simulation can give learners repeated practice with emergency scenarios. Prior work has linked the experience of the team managing the circuit to patient outcomes, but our data cannot show that improved comfort translates into better care. We did not evaluate patient outcomes in relation to training, and future work should link educational measures to clinical outcomes such as complications and survival.

Challenges and future directions

A central challenge is the retention of skills, which depends on each specialist’s exposure to ECMO cases. Between 2021 and 2025, PCH averaged approximately 55 ECMO circuits per year across three ICUs, a substantial volume by ELSO standards; exposure per specialist, however, depends on the size of the specialist pool. Quarterly patient care report conferences provide additional learning from cases. The high variability of patient conditions and the many factors that influence circuit management also make training difficult to standardize.

Future directions include virtual and augmented reality platforms for ECMO device training, which have begun to emerge and may allow learners to practice circuit priming and troubleshooting outside of the traditional water lab [12], and could help disseminate ECMO training to centers with varying case volumes.

Global collaboration and knowledge sharing are currently achieved through communities such as the ELSO, which has been in practice since 1989 [13]. ELSO also publishes guidelines for the training and continuing education of ECMO specialists that inform curricula such as the one described here.

Limitations

This study has several limitations. It was conducted at a single center, which may limit generalizability to institutions with different case volumes, staffing models, or patient populations. The comfort-level surveys relied on self-report rather than objective, observed competency measures, and were subject to potential response and social desirability bias given their anonymous but voluntary nature. Survey sample sizes for each training session were small (4 to 27 respondents per item, with fewer respondents in the fellows’ boot camp), which limits the precision of the reported estimates. The survey instrument was not validated, different items and response scales (comfort versus confidence) were used in the spring and fall sessions, the wording of some fellows’ items differed between pre- and post-surveys, and dichotomizing responses at 4 or 5 discards information. In the fellows’ boot camp, the number of respondents differed between pre- and post-surveys, and those who completed the post-survey may have been more engaged. Pre- and post-training surveys were not linked at the individual level, precluding paired analysis, and long-term retention of skills and knowledge beyond the immediate post-training period was not assessed. Finally, because skill retention depends on each specialist’s exposure to cases and was not assessed, the durability of any differences in self-reported comfort is unknown. All 95% CIs for pre- and post-training differences included zero, so the differences are descriptive and imprecise and should not be interpreted as evidence of an effect. We did not collect data on prior ECMO experience, professional role, or years in practice, and could not adjust for concurrent ECMO case exposure, staff turnover, or other factors that may influence self-reported comfort.

## Conclusions

In this single-center descriptive evaluation, self-reported comfort was numerically higher after sessions, with imprecise estimates; findings are descriptive and hypothesis-generating. Neither competence nor patient outcomes were measured. Future evaluations should use validated instruments, linked and longitudinal assessments, objective competency measures, and patient outcomes.
